# Narciclasine triggers apoptosis in osteosarcoma cells via JNK-mediated extrinsic and intrinsic pathways

**DOI:** 10.7150/ijms.128971

**Published:** 2026-02-11

**Authors:** Jia-Sin Yang, Chia-Hsuan Chou, Pei-Ni Chen, Yi-Hsien Hsieh, Chih-Hsin Tang, Shun-Fa Yang, Ko-Hsiu Lu

**Affiliations:** 1 Department of Medical Research, Chung Shan Medical University Hospital, Taichung, Taiwan.; 2 Institute of Medicine, Chung Shan Medical University, Taichung, Taiwan.; 3 Department of Pharmacology, School of Medicine, China Medical University, Taichung, Taiwan.; 4 Department of Medical Laboratory Science and Biotechnology, Asia University, Taichung, Taiwan.; 5 Chinese Medicine Research Center, China Medical University, Taichung, Taiwan.; 6 Department of Orthopedics, Chung Shan Medical University Hospital, Taichung, Taiwan.; 7 School of Medicine, Chung Shan Medical University, Taichung, Taiwan.

**Keywords:** narciclasine, osteosarcoma, apoptosis, caspase, JNK pathway

## Abstract

Osteosarcoma, the most prevalent bone cancer in teenagers, constitutes 3-5% of pediatric cancers and carries a poor prognosis due to its high metastatic potential. Although narciclasine induces apoptosis in various cancers, its effects on human osteosarcoma cells remain unclear. This study investigated the apoptotic impact of narciclasine on U2OS and HOS osteosarcoma cells. It was found that narciclasine induces dose-dependent apoptosis and sub-G_1_ phase cell cycle arrest. Human apoptosis array and Western blot analyses showed reduced inhibitors of apoptosis 1 (cIAP-1) and survivin expression while increasing cleaved caspases (8, 9, and 3) and PARP. Narciclasine activated extracellular signal-regulated protein kinases (ERK)1/2, c-Jun N-terminal kinases (JNK)1/2, and p38 phosphorylation. However, the JNK inhibitor (JNK-IN-8) suppressed the narciclasine-induced increase in cleaved caspase expression and apoptosis, whereas ERK and p38 inhibitors had no effect. These findings highlight the central role of JNK signaling in mediating extrinsic and intrinsic apoptotic pathways in osteosarcoma cells treated with narciclasine.

## Introduction

Narciclasine, also referred to as lycoricidinol, is a polyhydroxy alkaloid classified under isocarbostyril alkaloids 1-3. The natural compound is secreted by *Narcissus* species, including daffodils. First isolated in 1967 from the mucus of *Narcissus* L. bulbs of the Amaryllidaceae (amaryllis) family, it is a potent plant growth inhibitor 3. Since the Middle Ages, these flowering plants have been used in traditional medicine to treat cancer in various cultures, including Central America, North Africa, Arabia, and China 3. Narciclasine exhibits diverse bioactive properties, such as strong anti-inflammatory, anti-angiogenic, and antiviral effects 1, 4. Furthermore, it has shown remarkable anticancer activity against various malignancies, including brain, oral, breast, gastric, colon, and non-small cell lung cancers 2, 5-10. These diverse bioactive properties highlight its potential as a promising candidate for further therapeutic exploration.

Osteosarcoma, the most common primary malignant bone tumor, accounts for 35% of malignant bone tumors and 3-5% of pediatric cancers 11, 12. Predominantly affecting teenagers, with a secondary peak in older adults, 80-90% of cases arise in long bones, mainly in the metaphysis, with 85% involving the distal femur, proximal tibia, and proximal humerus 12-14. Its poor prognosis is largely due to a high propensity for lung metastases 11, 15. Historically, survival rates were below 20% in the 1970s when treatment relied on surgery or amputation 16, 17. The advent of neoadjuvant chemotherapy improved outcomes, enabling limb preservation in over 90% of cases and 5-year survival rates of 60-75% for localized tumors 11, 12. However, survival plummets to 20% when pulmonary metastases are present at diagnosis, with lung metastases remaining the leading cause of mortality 11-13. Advances in imaging, such as PET/CT, PET/MRI, and dynamic MRI, have enhanced metastasis detection and chemotherapy response evaluation 18-20. Novel therapeutic agents are under investigation to address treatment failures and improve outcomes.

Apoptosis, a form of programmed cell death, is critical for development and tissue homeostasis 21. It is marked by distinct morphological and biochemical changes, including cytoplasmic and nuclear condensation, DNA fragmentation, cell shrinkage, and membrane blebbing while maintaining the integrity of the cytoplasmic membrane 21, 22. In cancer cells, such as osteosarcoma, apoptosis is regulated by complex interactions among various pathways, including the mitogen-activated protein kinase (MAPK)/extracellular signal-regulated kinase (ERK), c-Jun N-terminal kinase (JNK), and p38 pathways, which mediate stress-induced apoptotic signals 23-25. These pathways initiate caspase cascades through mechanisms such as the formation of the death-inducing signaling complex (DISC), involving the Fas receptor, Fas-associated death domain, and subsequent caspase-8 recruitment. The intrinsic pathway is triggered by stressors such as DNA damage, mitochondrial dysfunction, or growth factors withdrawal, leading to cytochrome c release from mitochondria, apoptosome formation, and caspase-9 activating 21. The extrinsic and intrinsic pathways converge on effector caspases, such as caspase-3, which execute apoptosis. This convergence is finely tuned by inhibitors of apoptosis proteins (IAPs), such as cellular IAP (cIAP)-1 and survivin, ensuring precise control over the apoptotic process 26.

Most current cancer therapies focus on inducing apoptosis, as its failure can lead to treatment resistance. Understanding apoptotic mechanisms, including caspase cascades and the intrinsic and extrinsic pathways, is crucial for developing targeted therapies for metastatic osteosarcoma 3. Although narciclasine has shown antiproliferative, apoptotic, and anti-invasive effects in various cancers 2, 5-8, its impact on osteosarcoma remains unknown. This study aims to assess its anticancer efficacy and explore underlying mechanisms via MAPK pathways in human osteosarcoma.

## Materials and Methods

### Cell culture and narciclasine treatment

Human osteosarcoma U2OS (derived from a 15-year-old female) and HOS (from a 13-year-old female) cells were obtained from the Food Industry Research and Development Institute (Hsinchu, Taiwan). U2OS cells were cultured in Dulbecco's Modified Eagle Medium (DMEM, Gibco, NY, USA), and HOS cells were cultured in Eagle's minimum essential medium (MEM; Gibco, NY, USA). Both media were supplemented with 10% fetal bovine serum (FBS; Gibco, 26140-079, NY, USA), 1% penicillin (100 U/mL)/streptomycin (100 μg/mL) (Gibco, NY, USA), and 5 mL NEAA (Gibco, NY, USA). U2OS and HOS cells were plated in 24-well plates and treated with various concentrations (0.5, 1, 2, and 4 μM) of narciclasine (Sigma-Aldrich, SML2805) for 6 or 24 hours for the subsequent assays. Cells were maintained in a humidified incubator with 5% CO2 at 37℃, as previously described 17, 18.

### Cell viability analysis; Microculture tetrazolium (MTT) assay

U2OS (8.0 × 10^4^ cells/well) and HOS (8.0 × 10^4^ cell/well) cells were cultured in 24-well plates and treated with narciclasine (0, 0.5, 1, 2, and 4 μM) at 37℃ for 24 hours. Cytotoxicity at varying concentrations of narciclasine was assessed using the MTT (3-(4,5-dimethylthiazol-2-yl)-2,5-diphenyltetrazolium bromide) colorimetric assay, as previously described 27, 28. The viable cell count was determined spectrophotometrically at 563 nm by measuring formazan production after isopropanol solubilization.

### Cell-cycle and apoptosis analysis

Flow cytometry was used to analyze cellular DNA content and cell counts for cell cycle and apoptosis in U2OS (8.0 × 10^5^ cells/dish) and HOS (8.0 × 10^5^ cells/dish) cells treated with narciclasine (0, 0.5, 1, 2, and 4 μM) at 37℃ for 24 hours in 6-cm dishes. Cells were fixed in 70% ethanol overnight, resuspended in PI/RNase Staining Buffer (BD Biosciences, San Jose, CA, USA), incubated at RT for 15 minutes, and analyzed with a flow cytometer (Accuri C6 plus, Becton-Dickinson, San Jose, CA, USA) equipped with a 488 nm argon-ion laser, as previously described 29, 30.

Annexin V, a fluorescent protein that binds to phosphatidylserine, detects early apoptosis by marking membrane phospholipid translocation prior to DNA breakdown, offering an advantage over PI staining. U2OS (8.0 × 10^5^ cells/dish) and HOS (8.0 × 10^5^ cells/dish) cells were treated with narciclasine (0, 0.5, 1, 2, and 4 μM) at 37℃ for 24 hours in 6-cm dishes. After trypsinization, the Annexin V-fluorescein isothiocyanate-labeled (FITC) Apoptosis Detection Kit I (BD Biosciences, San Jose, CA, USA) was used according to the manufacturer's instructions. Flow cytometry with PI staining was employed to analyze the cell cycle and differentiate apoptosis from necrosis, as previously described 29, 30.

### Human apoptosis array

Protein lysates from U2OS (2.4 × 10^6^ cells/dish) and HOS (2.4 × 10^6^ cells/dish) cells treated with DMSO or 4 μM narciclasine at 37℃ for 24 hours in 10-cm dishes were analyzed using a Human Apoptosis Array Kit (R&D Systems, Minneapolis, MN, USA) to explore apoptosis mechanisms. The kit simultaneously detected 35 apoptosis-related proteins. Captured proteins on nitrocellulose membranes were visualized using biotinylated detection antibodies and chemiluminescent reagents, following the manufacturer's standard protocols 31, 32.

### Preparation of cell lysates and Western blotting

To investigate the molecular mechanisms, U2OS (8.0 × 10^5^ cells/dish) and HOS (8.0 × 10^5^ cells/dish) cells were cultured in 6-cm plates at 37℃ for 24 hours and treated with narciclasine (0, 0.5, 1, 2, and 4 μM) for 6 or 24 hours. Total cell lysates were then prepared for Western blot analysis using specific primary antibodies targeting caspases (3, 8, and 9), cIAP-1, surviving, cleaved caspases (3, 8, and 9), phosphorylated and unphosphorylated ERK1/2, JNK1/2, p38, and poly adenosine diphosphate-ribose polymerase (PARP) (Cell Signaling Technology, Danvers, MA, USA). The blots were incubated with HRP-conjugated secondary antibodies, and band intensities were quantified by densitometry, as previously described 33, 34.

### Statistical analysis

Values are presented as mean ± standard deviation (SD). Comparisons among three or more groups were conducted using one-way ANOVA, followed by Tukey's post-hoc test for equal sample sizes or Scheffe's test for unequal sample sizes. All experiments were performed in triplicate and repeated independently at least three times. Statistical significance was set at p < 0.05.

## Results

### Narciclasine displays cytotoxicity towards human osteosarcoma U2OS and HOS cells

**Figure 1:**
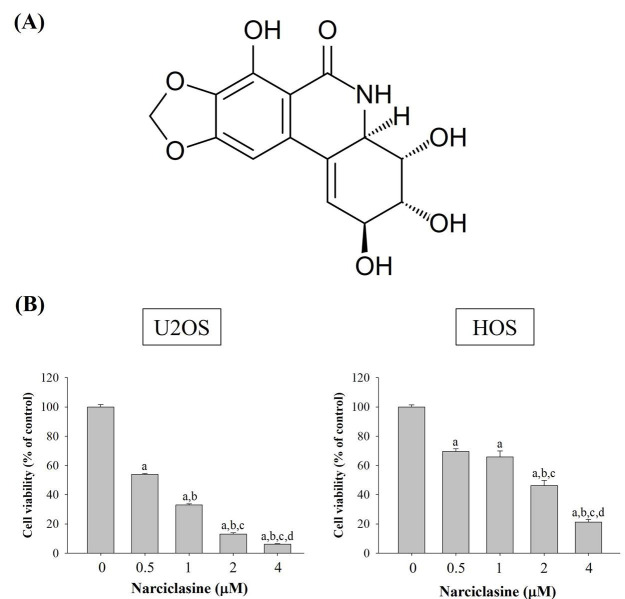
** Narciclasine inhibits the proliferation of U2OS and HOS cells.** (A) The chemical structure of narciclasine is shown. (B) U2OS and HOS cells were treated with increasing concentrations of narciclasine (0, 0.5, 1, 2, and 4 μM) for 24 hours. Cell viability was assessed using the MTT assay, and the effects were quantified and presented as mean ± S.D. All experiments were performed in three independent experiments. Statistical analysis was performed using ANOVA with Scheffe's post hoc comparison. For U2OS: *n* ≥ 4, *F* = 7860.132, *p* < 0.001. For HOS: *n* ≥ 4, *F* = 485.907, *p* < 0.001. ^a^ Significantly different, *p* < 0.05, when compared to control. ^b^ Significantly different, *p* < 0.05, when compared to 0.5 μM. ^c^ Significantly different, *p* < 0.05, when compared to 1 μM.^ d^ Significantly different, *p* < 0.05, when compared to 2 μM.

To evaluate the efficacy of narciclasine (Figure 1A) against human osteosarcoma, its cytotoxicity was assessed in U2OS and HOS cells using an MTT assay. In both cell lines, viability was significantly reduced compared to controls (0 μM) at narciclasine concentrations of 0.5, 1, 2, and 4 μM after 24 hours (p < 0.001 for both) (Figure 1B). A clear dose-dependent reduction in proliferation was observed. Specifically, treatment with 1 μM narciclasine reduced U2OS cell viability by more than 60%, and 4 μM caused a 90% reduction. Similarly, HOS cells showed a 50% decrease in viability at 2 μM and 70% at 4 μM.

### Narciclasine induces cell-cycle arrest in sub-G1 phase and apoptosis in U2OS and HOS cells

**Figure 2:**
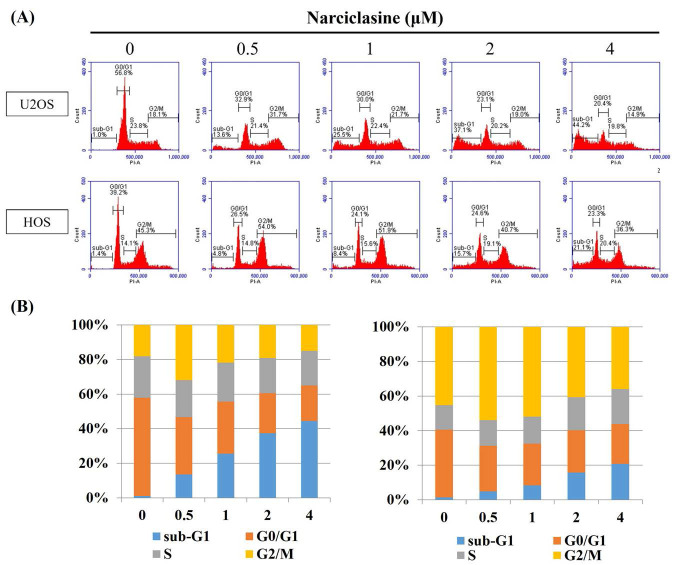
** Narciclasine arrests cell cycle progression in U2OS and HOS.** (A) U2OS and HOS cells were explored to increasing concentrations of narciclasine (0, 0.5, 1, 2, and 4 M) for 24 hours. The cells were then stained with PI and analyzed using flow cytometry to assess cell cycle distribution. (B) The resulting cell cycle profiles were quantified and presented for comparative analysis.

Flow cytometry was employed to analyze cell cycle distribution (G0/G1, S, G2/M) and investigate the cytotoxic mechanism of narciclasine in U2OS and HOS cells. Following 24-hour treatment with narciclasine (0.5, 1, 2, and 4 μM), a significant increase in the sub-G1 fraction was observed, rising from 13.6% to 44.2% in U2OS cells (control: 1.0%) and from 4.8% to 21.1% in HOS cells (control: 1.4%) (Figure 2A-B). These results indicate that the cytotoxic effects of narciclasine are associated with cell cycle arrest in the sub-G1 phase. Furthermore, an increase in the G2/M phase fraction was detected at lower concentrations (0.5 and 1 μM), followed by a decrease at higher concentrations (2 and 4 μM) in both cell lines.

### Narciclasine induces apoptosis in U2OS and HOS cells

**Figure 3:**
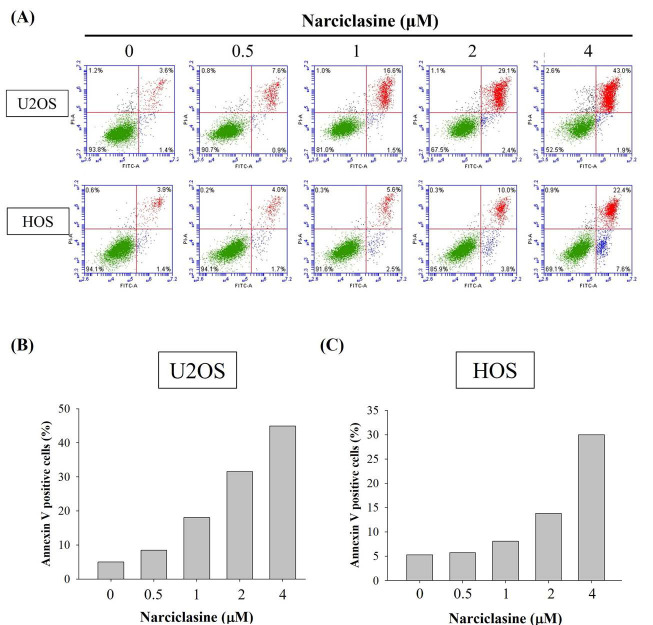
** Narciclasine induces apoptosis in U2OS and HOS cells.** (A) U2OS and HOS cells were treated with increasing concentrations of narciclasine (0, 0.5, 1, 2, and 4 M) for 24 hours. The cells were stained with Annexin V-FITC/PI and analyzed by flow cytometry to assess apoptosis. Viable cells were identified as negative for both FITC Annexin V and PI. Early apoptotic cells were positive for FITC Annexin V but negative for PI, while cells positive for both FITC Annexin V and PI were classified as being in late apoptosis or already dead. (B-C) Quantitative analysis combined early and late apoptotic cells to distinguish apoptotic processes from necrosis, providing a comprehensive overview of the apoptotic response.

To determine whether narciclasine suppresses cell proliferation through apoptosis rather than necrosis, an Annexin V-FITC/PI apoptosis assay was performed on U2OS and HOS cells. After 24 hours of treatment with narciclasine (0.5, 1, 2, and 4 μM), flow cytometry revealed significant increases in early apoptotic cells (Annexin V-FITC positive, PI negative) and late apoptotic cells (Annexin V-FITC positive, PI positive) (Figure 3A). These results demonstrate a substantial rise in both early and late apoptotic populations induced by narciclasine, aligning with the observed accumulation in the sub-G1 fraction in both cell lines (Figure 3B-C). Therefore, narciclasine effectively reduces cell viability and significantly induces apoptosis in human osteosarcoma U2OS and HOS cells.

### Narciclasine increases cleaved caspase-3 but decreases cIAP-1 and survivin in U2OS and HOS cells

**Figure 4:**
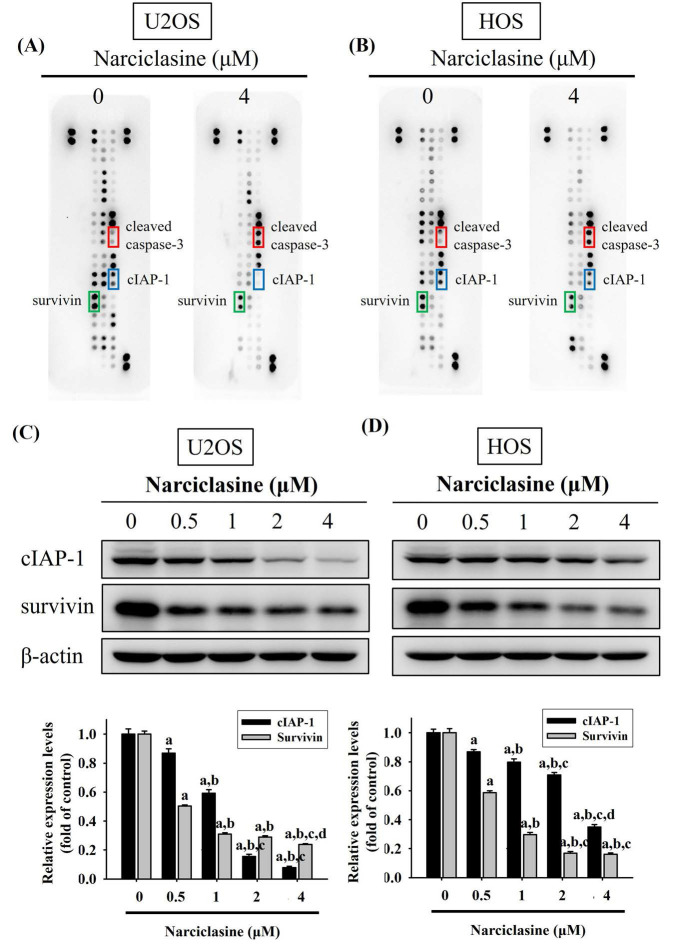
** Narciclasine modulates apoptosis-related proteins in U2OS and HOS cells.** (A) U2OS and (B) HOS cells were treated with narciclasine (0 and 4 μM) for 24 hours, and the expression of 35 apoptosis-related proteins was assessed using a human apoptosis array. (C-D) After treatment with increasing concentrations of narciclasine (0, 0.5, 1, 2, and 4 M) for 24 hours, two decreased levels of cIAP-1 and survivin, two proteins that showed significant reductions, were analyzed by Western blotting. Results are presented as mean ± S.D., with *n* = 3. ANOVA analysis with Tukey's posteriori comparison was performed. U2OS: cIAP1: *F* = 570.813, *p* < 0.001. survinin: *F* = 1409.006, *p* < 0.001; HOS: cIAP1: *F* = 301.741, *p* < 0.001. survinin: *F* = 832.521, *p* < 0.001. ^a^ Significantly different, *p* < 0.05, when compared to control. ^b^ Significantly different, *p* < 0.05, when compared to 0.5 μM. ^c^ Significantly different, *p* < 0.05, when compared to 1 μM. ^d^ Significantly different, *p* < 0.05, when compared to 2 μM.

A human apoptosis array was used to analyze apoptosis-related proteins and explore the mechanisms underlying narciclasine-induced apoptosis in U2OS and HOS cells. Treatment with 4 μM narciclasine for 24 hours significantly increased cleaved caspase-3 levels while decreasing cIAP-1 and survivin proteins, indicating their role as executioners in these cells (Figure 4A-B). These reductions in cIAP-1 and survivin treated with narciclasine were further validated by Western blotting, showing a dose-dependent effect in both U2OS (p < 0.001 for both) and HOS cells (p < 0.001 for both) (Figure 4C-D).

### Narciclasine activates both extrinsic and intrinsic apoptotic processes in U2OS and HOS cells

**Figure 5:**
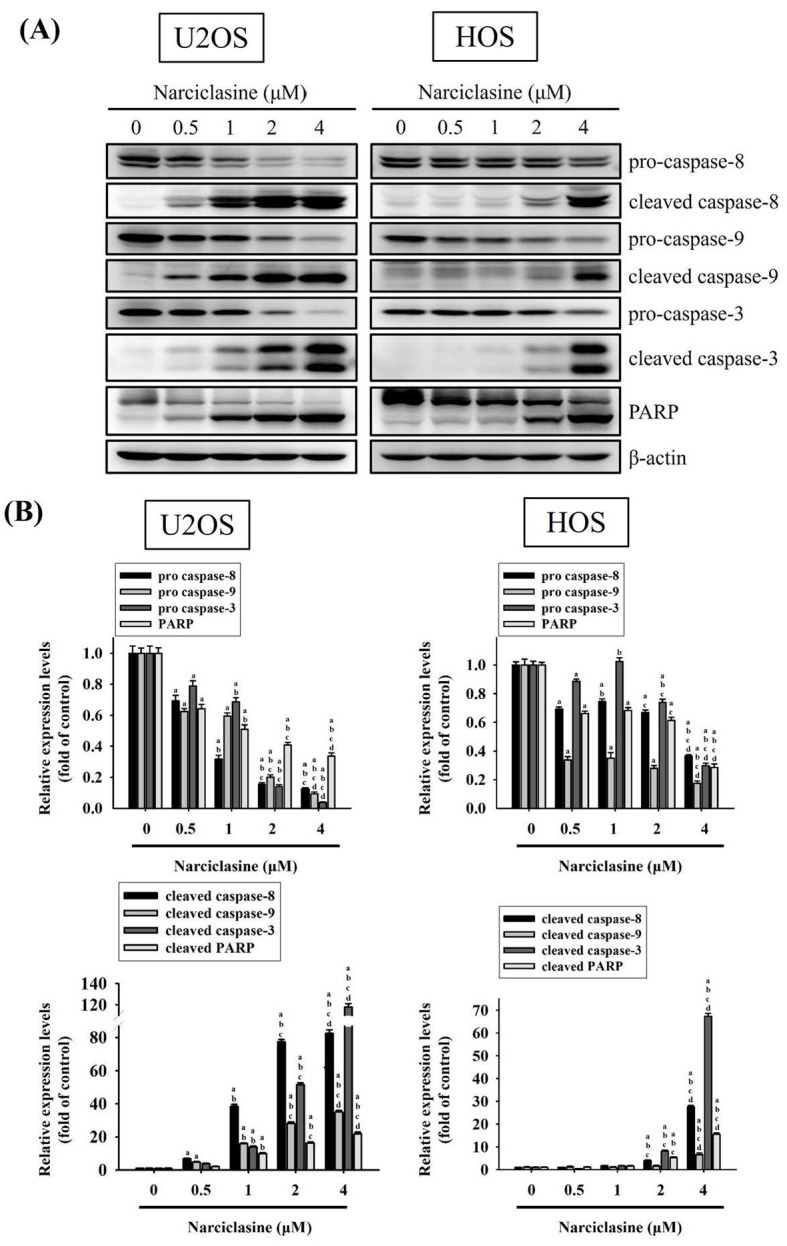
** Narciclasine activates caspases and PARP in U2OS and HOS cells.** (A) U2OS and HOS cells were treated with increasing concentrations of narciclasine (0, 0.5, 1, 2, and 4 M) for 24 hours. The relative expression levels of caspases 3, 8 and 9, their cleaved forms, and PARP were assessed through Western blot analysis. (B) Quantitative analysis of the Western blot results was performed. Data are presented as mean ± S.D., with *n* = 3. ANOVA analysis with Tukey's posteriori comparison was performed. U2OS: pro-caspase 8: *F* = 489.435, *p* < 0.001; pro-caspase 9: *F* = 891.180, *p* < 0.001; pro-caspase 3: *F* = 627.381, *p* < 0.001; PARP: *F* = 290.082, *p* < 0.001; cleaved caspase 8 (43/41 kDa): *F* = 2497.977, *p* < 0.001; cleaved caspase 9 (37/35 kDa): *F* = 1611.113, *p* < 0.001; cleaved caspase 3 (19/17 kDa): *F* = 2938.711, *p* < 0.001; cleaved PARP: *F* = 629.213, *p* < 0.001; HOS: pro-caspase 8: *F* = 547.997, *p* < 0.001; pro-caspase 9: *F* = 385.105, *p* < 0.001; pro-caspase 3: *F* = 551.551, *p* < 0.001; PARP: *F* = 464.187, *p* < 0.001; cleaved caspase 8 (43/41 kDa): *F* = 3945.413, *p* < 0.001; cleaved caspase 9 (37/35 kDa): *F* = 87.666, *p* < 0.001; cleaved caspase 3 (19/17 kDa): *F* = 6814.416, *p* < 0.001; cleaved PARP: *F* = 842.322, *p* < 0.001. ^a^ Significantly different, *p* < 0.05, when compared to control. ^b^ Significantly different, *p* < 0.05, when compared to 0.5 μM. ^c^ Significantly different, *p* < 0.05, when compared to 1 μM. ^d^ Significantly different, *p* < 0.05, when compared to 2 μM.

Western blotting confirmed the effects of narciclasine on the caspase cascade in U2OS and HOS cells. Treatment with narciclasine (0.5, 1, 2, and 4 μM) for 24 hours resulted in dose-dependent increases in cleaved caspase-8 (43/41 kDa), caspase-9 (37/35 kDa), caspase-3 (19/17 kDa), and cleaved PARP (89 kDa) (U2OS: p < 0.001 for all; HOS: p < 0.001 for all) (Figure 5A-B). Concurrently, the levels of pro-caspase-8 (57 kDa), pro-caspase-9 (47 kDa), pro-caspase-3 (32 kDa), and PARP (116 kDa) decreased in a dose-dependent manner. These results indicate that narciclasine induces apoptosis in both cell lines by activating the extrinsic caspase-8 and intrinsic caspase-9 pathways, leading to the activation of effector caspase-3.

### Narciclasine triggers extrinsic and intrinsic apoptotic cascades via JNK signaling in U2OS and HOS cells

**Figure 6:**
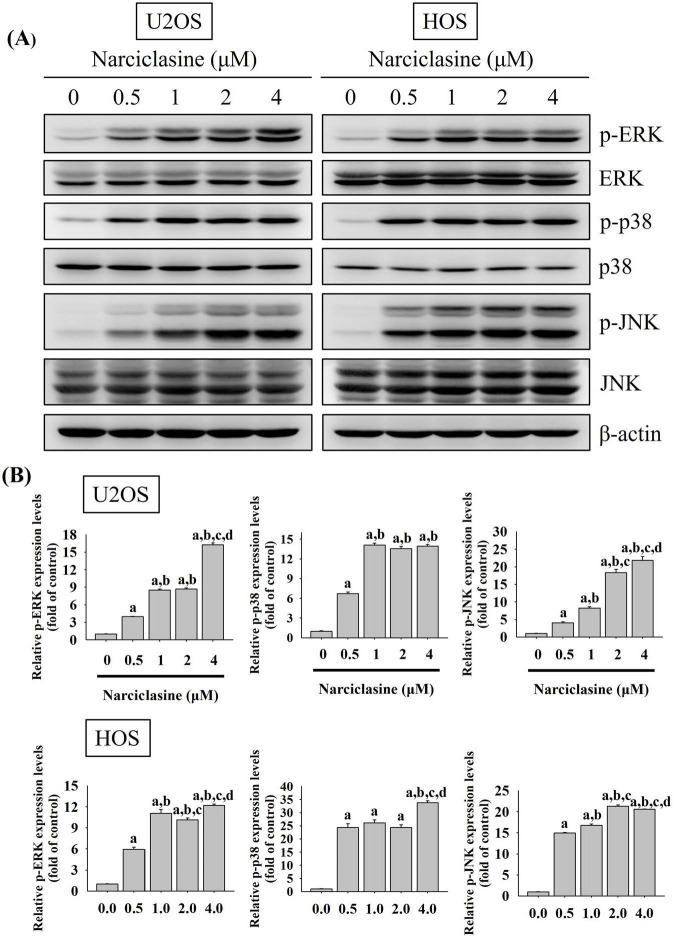
** Narciclasine modulates phosphorylation of ERK1/2, JNK1/2, and p38 in U2OS and HOS cells.** (A) U2OS and HOS cells were treated with increasing concentrations of narciclasine (0, 0.5, 1, 2, and 4 M) for 6 hours. The relative expression levels of ERK1/2, JNK 1/2, and p38, along with their phosphorylated forms, were analyzed by Western blotting. (B) The Western blot results were quantitatively analyzed. Data are presented as mean ± S.D., with *n* = 3. ANOVA analysis with Tukey's posteriori comparison was performed. U2OS: p-ERK/ERK: *F* = 2754.492, *p* < 0.001; p-JNK/JNK: *F* = 574.223, *p* < 0.001; p-p38/p38: *F* = 1717.561, *p* < 0.001. HOS: p-ERK/ERK: *F* = 623.477, *p* < 0.001; p-JNK/JNK: *F* = 4248.166, *p* < 0.001; p-p38/p38: *F* = 413.783, *p* < 0.001. ^a^ Significantly different, *p* < 0.05, when compared to control. ^b^ Significantly different, *p* < 0.05, when compared to 0.5 μM. ^c^ Significantly different, *p* < 0.05, when compared to 1 μM. ^d^ Significantly different, *p* < 0.05, when compared to 2 μM.

Mitogen-activated protein kinases (MAPKs) play a central role in transducing signals that regulate apoptotic responses under diverse stress conditions 35, 36. In osteosarcoma, aberrant activation of MAPK signaling pathways has been closely associated with tumor development, disease progression, and reduced sensitivity to therapeutic interventions 37, 38. Given the critical involvement of MAPK signaling in osteosarcoma pathogenesis, we next examined whether Narciclasine modulates MAPK pathway activation in osteosarcoma cells. Narciclasine dose-dependently increased the phosphorylation of ERK1/2, JNK1/2, and p38 in U2OS and HOS cells (U2OS: p < 0.001 for all; HOS: p < 0.001 for all) (Fig. 6A-B), indicating narciclasine-induced activation of these pathways. To further explore the signaling pathways involved in apoptotic cascades, specific inhibitors targeting ERK1/2 (U0126), JNK1/2 (JNK-IN-8), and p38 (SB203580) were used to assess their effects on narciclasine-induced activation of cleaved caspases (8, 9, and 3) in U2OS and HOS cells.

**Figure 7:**
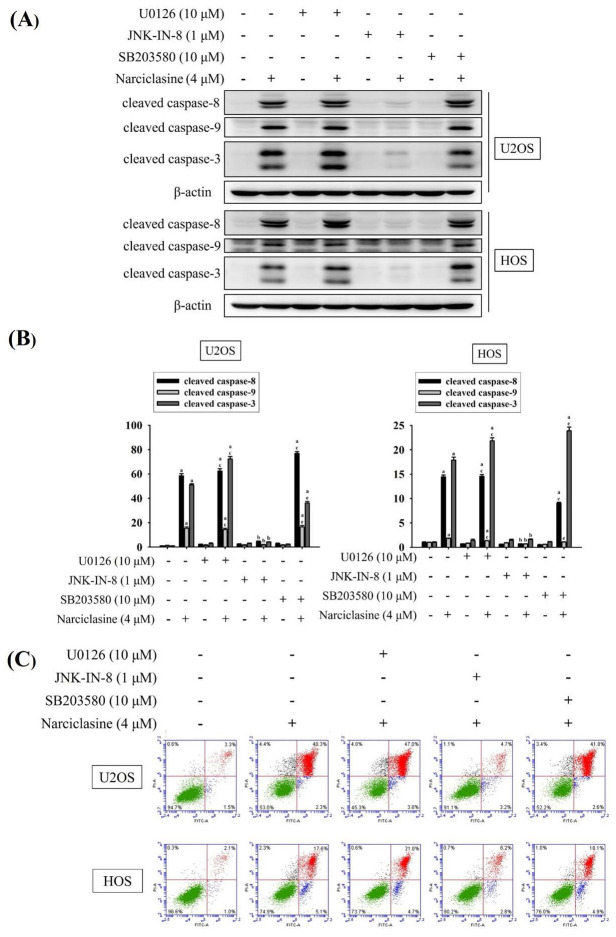
** Effects of narciclasine and ERK1/2, JNK1/2, and p38 inhibitors on cleaved caspase expression in U2OS and HOS cells.** U2OS and HOS cells were pretreated with or without specific inhibitors—10 μM of U0126 (ERK1/2 inhibitor), 1 μM of JNK-in-8 (JNK1/2 inhibitor), and 10 μM SB203580 (p38 inhibitor) for 2 hour. They were followed by treated with 0 or 4 μM narciclasine for an additional 24 hours, (A) The expression levels of cleaved caspases 3, 8, and 9 were analyzed using Western blotting. (B) The Western blot results were quantitatively analyzed. Data are expressed as mean ± S.D., with *n* = 3. ANOVA analysis with Tukey's posteriori comparison was performed. U2OS: cleaved caspase 8 (43/41 kDa): *F* = 2821.995, *p* < 0.001; cleaved caspase 9 (37/35 kDa): *F* = 257.284, *p* < 0.001; cleaved caspase 3 (19/17 kDa): *F* = 2905.593, *p* < 0.001. HOS: cleaved caspase 8 (43/41 kDa): *F* = 2994.330, *p* < 0.001; cleaved caspase 9 (37/35 kDa): *F* = 140.512, *p* < 0.001; cleaved caspase 3 (19/17 kDa): *F* = 1818.473, *p* < 0.001. ^a^ Significantly different, *p* < 0.05, when compared to control. ^b^ Significantly different, *p* < 0.05, when compared to 4 μM narciclasine. ^c^ Significantly different, *p* < 0.05, when compared to U0126. ^d^ Significantly different, *p* < 0.05, when compared to JNK-in-8. ^e^ Significantly different, *p* < 0.05, when compared to SB203580. (C) Flow cytometry analysis was performed to assess the effects of MAPK inhibitors on narciclasine-induced apoptosis using Annexin V-FITC/PI staining.

Treatment with 4 μM narciclasine activated cleaved caspases (8, 9, and 3) (U2OS: p < 0.05 for all; HOS: p < 0.05 for all) (Figure 7A-B). Notably, JNK-IN-8 significantly inhibited the narciclasine-induced activation of cleaved caspases (8, 9, and 3) (U2OS: JNK-IN-8: p < 0.05 for all; HOS: JNK-IN-8: p < 0.05 for all), whereas U0126 and SB203580 did not prevent these effects. These findings highlight the critical role of JNK signaling in the activation of both extrinsic initiator caspase-8 and intrinsic initiator caspase-9, leading to the subsequent activation of effector caspase-3 in U2OS and HOS cells.

Using flow cytometry, we further confirmed that JNK signaling mediates the activation of both extrinsic and intrinsic apoptotic pathways, leading to apoptosis. Treatment with 4 μM narciclasine significantly induced cellular apoptosis in U2OS and HOS cells (Figure 7C). Notably, JNK-IN-8 markedly reduced narciclasine-induced apoptosis in both cell lines, whereas U0126 and SB203580 had no such effect. These results corroborate previous findings, demonstrating that narciclasine induces apoptosis in U2OS and HOS cells via activation of both the extrinsic (caspase-8) and intrinsic (caspase-9) pathways, ultimately leading to the activation of the downstream effector caspase-3.

## Discussion

Apoptosis can be triggered through either the extrinsic (death receptor) or intrinsic (mitochondrial) pathways, both of which converge on the activation of initiator and effector caspases 21, 26, 39. Through a series of experiments, we found that narciclasine induces apoptosis via both pathways by activating the caspase cascade, downregulating cIAP-1 and survivin, and promoting the phosphorylation of ERK1/2, JNK1/2, and p38. Notably, pretreatment with a JNK-specific inhibitor significantly suppressed the cleavage of caspases 8, 9, and 3, as well as apoptosis, whereas inhibitors of ERK1/2 and p38 had no such effect. These results underscore the pivotal role of the JNK signaling pathway in mediating narciclasine-induced apoptosis in osteosarcoma U2OS and HOS cell lines, distinguishing it from the ERK and p38 pathways.

Narciclasine primarily exerts cytostatic effects in both plant and human cells at its IC50 concentration 40, with cancer cells being more sensitive than normal cells. As a novel topoisomerase I inhibitor, narciclasine exhibits potent anticancer activity at lower concentrations (~nM), selectively targeting cancer cells while sparing normal cells by arresting the cell cycle at the G2/M phase and inducing cell apoptosis 41. A concentration of 1 μM represents a pharmacological dose, 10-20 times higher than the IC50 of narciclasine 7. The actin cytoskeleton is crucial for cell proliferation, migration, and metastasis 4, 42. Narciclasine has been shown to modulate autophagy-dependent apoptosis in gastric cancer by inhibiting Akt/mTOR phosphorylation 2. At higher concentrations (≥1 μM), narciclasine selectively induces apoptosis in certain human cancer cell types through DISC activation and caspase-8 cleavage while sparing normal human fibroblasts 6. The downstream activation of effector caspases, such as caspase-3, is cell type-specific 6, 21. In prostate cancer PC-3 cells, narciclasine directly activates effector caspases via DISC assembly. Conversely, in breast cancer MCF-7 cells, narciclasine induces apoptosis by facilitating Bid processing, cytochrome c release, and apoptosome formation 6.

In colon carcinoma cells, narciclasine induces cell apoptosis by reducing mitochondrial membrane potential and Bcl-2 expression while increasing Bax, cleaved caspases 8/9/3, and cytoplasmic cytochrome c. It also suppresses the IL-17A/Act1/TRAF6/NF-κB pathway to promote apoptosis 8. Additionally, narciclasine inhibits oral cancer metastasis by modulating the cathepsin B and ERK pathways 5, and exhibits preferential cytotoxicity in primary effusion lymphoma by arresting cell-cycle progression 1. In brain cancer, it targets guanosine triphosphate hydrolase, activates Rho and stress fiber formation in glioblastoma multiforme cells, and extends survival in preclinical models, though no patient cures have been reported 7, 42.

At pharmacological doses (1 μM in vitro, approximately 10 mg/kg in vivo), narciclasine exhibits strong proapoptotic and cytotoxic effects, demonstrating significant anticancer activity but with severe toxicity 7. In contrast, at physiological doses (50 nM in vitro, approximately 1 mg/kg in vivo), narciclasine shows cytostatic effects, displaying potent anti-metastatic activity in brain and oral cancer models without inducing toxicity 5, 7. These cytostatic effects are attributed to the disruption of actin cytoskeleton organization by targeting GTPases such as RhoA and elongation factor eEF1A, as well as regulating cathepsin B and ERK pathways, respectively. Consistent with earlier findings 43, narciclasine in this study at 4 µM arrested cell-cycle progression in the sub-G1 phase and induced apoptosis in osteosarcoma cells. This was accompanied by activating caspases 8/9/3, cleaving PARP, and increasing surface Annexin-V expression.

In this study, narciclasine, at lower pharmacological concentrations (0.5 and 1 μM), narciclasine increased the G2/M phase fraction in both cell lines while decreasing this friction at higher concentrations (2 and 4 μM). Although these effects may vary depending on the specific cell lines, cancer types, or concentrations examined 12, 44, 45, the findings align with previous research on crinane alkaloids, which are differentiated from lycorine alkaloids 43. For instance, haemanthamine and haemanthidine—two common α-crinanes from the Amaryllidaceae family—have been shown to increase G1 and G2/M phase fractions at 5 μM. Similarly, distichamine, a β-crinane, induced a concentration-dependent increase in sub-G1 DNA content (indicative of apoptotic cells), reaching 23.7% at the highest tested dose of 20 μM. Together, our results suggest that narciclasine, at pharmacological concentrations, shows potential as an effective agent for inducing sub-G1 phase arrest and apoptosis while arresting G2/M at physiological levels in human osteosarcoma cells.

In conclusion, our study examined the anticancer effects of narciclasine in human osteosarcoma cells, highlighting its role in inducing apoptosis via the JNK signaling pathway in U2OS and HOS cell lines, distinct from ERK and p38 pathways. Further research is needed to validate these findings *in vivo* and assess the therapeutic potential of narciclasine in clinical trials for human osteosarcoma. Future studies may focus on exploring novel aspects of narciclasine as an adjuvant therapy to broaden treatment options and improve outcomes.
